# Mutations in the H7 HA and PB1 genes of avian influenza a viruses increase viral pathogenicity and contact transmission in guinea pigs

**DOI:** 10.1080/22221751.2019.1663131

**Published:** 2019-09-10

**Authors:** Carola Dreier, Patricia Resa-Infante, Swantje Thiele, Stephanie Stanelle-Bertram, Kerstin Walendy-Gnirß, Thomas Speiseder, Annette Preuss, Zacharias Müller, Hans-Dieter Klenk, Jürgen Stech, Gülsah Gabriel

**Affiliations:** a Viral Zoonosis -One Health, Heinrich Pette Institute, Leibniz Institute for Experimental Virology, Hamburg, Germany; b Institute of Virology, University of Veterinary Medicine, Hannover, Germany; c Institute for Virology, Philipps University of Marburg, Marburg, Germany; d Institute for Molecular Virology and Cell Biology, Friedrich-Loeffler-Institute, Greifswald, Germany; # Current address: University of Ulm, Ulm, Germany.; † Current address: IrsiCaixa AIDS Research Institute, Barcelona, Spain.

**Keywords:** H7 avian influenza A viruses, HA and PB1 gene mutations, pathogenicity, transmission, guinea pigs

## Abstract

Avian influenza A viruses (AIV) of the H7 subtype continue to evolve posing a pandemic threat. However, molecular markers of H7N7 AIV pathogenicity and transmission in mammals remain poorly understood. In this study, we performed a systematic *in vitro* and *in vivo* analysis by comparing an H7N7 highly pathogenic AIV and its ferret adapted variant. Passaging an H7N7 AIV in ferrets led to six mutations in genes encoding the viral polymerase complex and the viral surface proteins. Here, we show that mutations in the H7 hemagglutinin gene cause increased pathogenicity in mice. Contact transmission between guinea pigs required additional mutations in the gene encoding the polymerase subunit PB1. Thus, particular vigilance is required with respect to HA and PB1 mutations as predictive molecular markers to assess the pandemic risk posed by emerging H7 avian influenza viruses.

## Introduction

Emerging influenza A viruses (IAV) viruses continue to pose a major health burden. Herein, particularly avian H5 and H7 IAV pose an ongoing threat for animal and human health. Currently, avian IAV of the H7N9 subtype are classified by the World Health Organization (WHO) as posing the highest risk candidates to cause the next influenza pandemic.

Avian H7N9 IAV outbreaks were first reported in March 2013 in China and spread later to Hong Kong, Macau, and Taiwan [1–4]. Since then, 1567 human and 615 fatal cases have been reported [5]. Until 2017, H7N9 IAV comprised of low pathogenic avian influenza viruses (LPAIV) only, causing no or little symptoms in poultry. Thus, H7N9 LPAIV managed to spread silently and widely in China to many administrative regions [6]. This silent nature of H7 LPAIV to cause subclinical disease in poultry made surveillance efforts particularly challenging. H7 LPAIV infections with distinct clinical signs were recognized in humans after interspecies transmission. In 2016, H7 LPAIV had evolved to a highly pathogenic avian influenza virus (H7 HPAIV) now causing overt disease in humans and also in poultry. Compared to its H7 LPAIV precursor, the H7 HPAIV was even able to spread to previously unaffected provinces in mainland China leading to large outbreaks in chicken farms and continuous infections in humans [6]. The fifth wave of H7N9 infections (October 2016-September 2017) was the largest in terms of geographical distribution, number of human cases and outbreaks in poultry [6]. In contrast to the previous waves, the majority of circulating H7N9 strains in 2018 displayed the HPAIV phenotype [7].

The still evolving nature of H7N9 avian influenza viruses and their persistence to cause diseases in animal and man prompted the Chinese government to initiate a massive vaccination campaign. Since September 2017, all poultry in mainland China with focus on longer-lived chickens has been vaccinated against H5 and H7 strains [6]. More than a year after the massive vaccination campaign, in 2018, a significant drop in the number of H7N9 detections in avian species was reported by the national active surveillance efforts. Likewise, human H7N9 cases almost entirely vanished with only sporadic cases [5]. However, some post-vaccination H7N9 viruses were found to have further evolved into various genotypes, including new variants characterized by increased virulence in mice and ducks compared to the pre-vaccination H7N9 virus lineage [7–9]. Moreover, vaccination against H7N9 IAV also resulted in new H7N2 HPAIV variants with elevated virulence in mice likely due to altered selective pressure against H7 IAV [7]. Thus, circulating H7 avian influenza viruses in general demand continuous vigilance to their incidence in both wild birds and domestic poultry to assess their potential to become highly pathogenic to and transmittable among humans.

In this study, we sought to study molecular markers that mediate increased H7 HPAIV pathogenicity and transmissibility in mammals. Therefore, we initiated a comparative study using an H7N7 HPAIV (SC35) as a model strain that is highly virulent in chicken but not in mammals as well as its ferret-adapted successor (SC35F) [10,11]. Using mammalian models, we revealed and characterized novel molecular markers of virulence and transmissibility.

## Materials and methods

### Biosafety statement

All experiments using recombinant influenza A viruses were approved by the responsible German authorities (Behörde für Umwelt und Energie, Referat Gentechnik, Hamburg). The ferret-adapted H7 HPAIV SC35F was classified as a BSL-4 agent and was not used in experiments due to biosafety concerns. Herein, only recombinant viruses classified as BSL-3 pathogens were further investigated, such as single gene reassortants (SGRs) possessing single SC35F genes in the SC35 genetic background, SC35F containing a monobasic cleavage site as well as single point mutants (SPMs) with SC35F single mutations in the SC35 backbone. Isolation of the SC35F genetic material was performed in the BSL-4 laboratory of the Bernhard-Nocht Institute for Tropical Medicine in Hamburg, Germany. All other experiments including animal studies were performed in the BSL-3 laboratories of the Heinrich Pette Institute, Leibniz Institute for Experimental Virology in Hamburg, Germany.

### Cells and viruses

MDCK II (Madin-Darby canine kidney) and HEK293T (Human embryonic kidney) cells were cultured in MEM (Minimal Essential Medium; Sigma-Aldrich) or DMEM (Dulbeccós Modified Eaglés Medium; Sigma-Aldrich), respectively. Culture media were supplemented with 10% FBS (fetal bovine serum; Biochrom), 1% L-glutamine (Sigma-Aldrich) and 1% penicillin–streptomycin (Sigma-Aldrich). Cells were cultured at 37°C, 95% rH (relative humidity) and 5% CO_2_.

The SC35 virus is an H7N7 HPAIV that is lethal in chicken and low pathogenic in mice and ferrets as described before [10,11]. SC35F is the ferret-adapted variant of SC35 that is lethal in ferrets [11]. Recombinant SC35/SC35F SGR and SPM viruses were generated by reverse genetics using the pHW2000 plasmid system as described before [10]. The SC35 virus was grown in specific pathogen free 11 d embryonated chicken eggs (ValoBiomedia) as described before [10]. All recombinant SC35/SC35F SGR and SPM viruses, including the recombinant A/Netherlands/213/03 (H3N2) and A/Vietnam/11/94 (H5N1-HA_monobasic_) viruses were propagated on MDCK II cells. The full SC35 gene sequence is deposited in the GenBank database (accession nos. DQ266094-DQ266101) [10].

### Transfection and vectors

Transfections were performed using Lipofectamine 2000 (Invitrogen) according to the manufacturer’s instructions except for the cell–cell fusion assay. Vector constructs used were pHW2000-PB2_SC35_, -PB1_SC35_, -PA_SC35_, -HA_SC35_, -NP_SC35_, -NA_SC35_, -M_SC35_, -NS_SC35_ [10] and additional plasmids were generated by site directed mutagenesis: pHW2000-PB2_SC35F_, -PB1_SC35F_, -HA_SC35F_, -NA_SC35F_, -HA_111T_, -HA_146S_, -HA_340R_, SC35F-HA_monobasic_, pCAGGS-HA_SC35,_ pCAGGS-HA_SC35F_, pCAGGS-HA_111T,_ pCAGGS-HA_146S_ and pCAGGS-HA_340R_. pCAGGS plasmids were generated by subcloning the respective HA gene segments. The pGAL4-VP16 and pGal5-luc plasmids [12] were kindly provided by Thomas Stamminger, University Hospital Ulm, Germany. Additionally, we used pPol-I-NP-Luc-human and pRL-TK (Promega) to measure viral polymerase activity as described previously [10].

### Receptor binding assay

For determining receptor binding preferences of the viral hemagglutinin, a hemagglutination (HA) assay with modified turkey red blood cells (TRBCs) was conducted as described before [11,13,14]. Briefly, sialic acids (SAs) were removed from TRBCs (Charles River) by incubation with 50 mU neuraminidase *Vibrio cholerae* (VCNA; Roche) in 1x PBS (Sigma-Aldrich) including 8 mM calcium chloride for 1 h at 37°C (16). Resialylation with α2,3-linked SAs was performed by incubation at 37°C for 2 h with 6 mU of α2,3-sialyltransferase from *Pasteurella multocida* (Sigma-Aldrich), whereas resialylation with α2,6-linked SA was achieved by incubation with 38 mU α2,6-sialyltransferase from *Photobacterium damsela* (Sigma-Aldrich). To generate α2,3-linked SA TRBCs and α2,6-linked SA TRBCs, 1.5 mM cytidine-5′-monophospho-N-acetylneuraminic acid sodium salt (CMP, Sigma-Aldrich) was used. Upon washing, 0.5% dilutions of VCNA treated, modified and non-modified TRBCs were prepared in 1x PBS supplemented with 1% BSA (Sigma-Aldrich).

### HA stability using cell–cell fusion assay at different pH values

For analysis of IAV HA driven cell–cell fusion, 293T effector cells were seeded in 6-well plates at 2 × 10^5^ cells/well and 293T target cells in 48-well plates at 0.4 × 10^5^ cells/well. At 24 post seeding, 293T effector cells were transfected with either 2 µg empty pCAGGS plasmid or pCAGGS encoding for the respective HA in combination with 1 µg pGAL4-VP16 plasmid, which encodes the herpes simplex virus VP16 transactivator fused to the DNA binding domain of the Saccharomyces cerevisiae transcription factor GAL4. In parallel, 293T target cells were transfected with 200 ng pGal5-luc plasmid, which encodes the luciferase reporter gene under the control of a promoter containing five GAL4 binding sites. The cells were transfected using polyethylenimine (PEI) in OptiMEM medium (Thermo Fisher) and cell culture medium was exchanged to transfection medium (DMEM, 10% FBS, 1% L-Gln) at 8 h post transfection. At 24h post transfection, 293T effector cells were detached and diluted in 4 ml fresh transfection medium/well and 100 µl were added to target cells. After 8 h incubation, cells were exposed to cell culture medium at different pH values (pH 4.6–5.2) for 20 min at 37°C. Afterwards, medium was exchanged to transfection media for 24 h. Subsequently, 293T cells were lysed with passive lysis buffer (Promega) and firefly luciferase activity was determined at 72 h post transfection using Luciferase Assay System (Promega) according to the manufactureŕs instructions and measured in a Tristar LB 941 Luminometer (Berthold).

### Dual Luciferase Reporter Assay

To measure viral polymerase activity, 6 × 10^5^ HEK293T cells were seeded in 6-well plates and co-transfected with 0.5 µg pHW2000 vector constructs encoding PB2, PB1, PA and NP genes of SC35 and SC35F viruses, respectively. Additionally, reporter constructs pPol-I-NP-Luc (encoding firefly luciferase) and pRL-TK (Promega; encoding Renilla luciferase) were co-transfected for normalization [10]. As a background control, vRNPs were transfected omitting the PB2 subunit. Cells were lysed using passive lysis buffer (Promega) and luciferase activity was determined 24 h post transfection using Dual-Luciferase Reporter Assay System (Promega) according to the manufactureŕs instructions and measured in a Tristar LB 941 Luminometer (Berthold).

### 4-MU-NANA assay

To measure neuraminidase (NA) activity, 4-methylumbelliferyl *N-*acetylneuraminic acid (4-MU-NANA, Sigma-Aldrich) was used. Experiments were performed as described previously [10]. Here, we used 1, 2.5, 5, 7.5 µl or 10 µl of 2^3^ HAU SC35 (NA_328A_), and SC35F (NA_328S_) viruses, respectively. Then, viruses were incubated with 50 µl of 40 µM 4-MU-NANA solved in buffer (6.8 mM CaCl2, 0.85% NaCl and 0.02 M TrisHCl, pH 7.3). Incubation was performed for 30 min at 37°C. Reaction was stopped with 100 µl of 0.1 M glycine buffer containing 25% ethanol (pH 10.7). Fluorescence data were obtained using Tecan Safire 2 (Thermo Fisher Scientific). Results are shown in relative fluorescent units with 1 µl of 2^3^ HAU SC35 virus set to 100%.

### NA elution assay

NA activity was additionally measured by NA erythrocyte elution assay. Therefore, 100 µl of 2^3^ HAU virus was serially diluted 1:2 in 1x PBS (Sigma-Aldrich). After addition of 50 µl of 1% chicken erythrocytes diluted in 0.9% saline solution (NaCl; Sigma-Aldrich) to the virus dilutions, the v-bottom 96-well plates were incubated at 37°C. Elution of the erythrocytes was assessed at 30 min, 1, 2, 3, and 4 h of incubation.

### HA modelling

The HA sequence from an H7N7 HPAIV was downloaded via the website https://www.rcsb.org/ and modelled as a 3D-structure using the software PyMol Molecular Graphics System (PyMOL(TM) 1.7.4.5 Edu – Educational Product, Copyright (C) Schrodinger, LLC). The Protein Data Base ID is 4DJ6.

## Animal experiments

### Mice

Six-week-old female BALB/c mice were purchased from Charles River. Mice obtained from Charles River were housed under specific pathogen-free conditions at the animal facility of the Heinrich Pette Institute, Leibniz Institute for Experimental Virology, Hamburg, Germany. Mice were anesthetized intraperitoneally (i.p.) with ketamine (100 mg/kg) and xylazine (10 mg/kg). In narcosis, mice were inoculated intranasally (i.n.) with a 50 µl inoculum containing 10^1^–10^6^ plaque forming units (p.f.u.) of the respective SC35/SC35F recombinant virus diluted in 1x PBS. Control groups were mock inoculated with 1x PBS. Body weight and survival were monitored for 14 days post infection (d p.i.). Additionally, on days 3 and 6 p.i., three mice of each group were sacrificed and organs were harvested for immunohistochemical analysis, determination of viral organ loads and cytokine/chemokine levels. Viral organ titers were determined via plaque assay in MDCK II cells and cytokine/chemokine levels using the Mouse Cytokine Array C1, RayBiotech Inc. as described before [14]. To assess the mouse lethal dose 50 (MLD_50_), mice were infected with serial dilutions and the MLD_50_ was calculated using the Reed-Muench method as described before [10].

### Guinea pigs

Hartley guinea pigs were obtained from Charles River and housed in special airflow chambers in the BSL-3 facility of the Heinrich Pette Institute, Leibniz Institute for Experimental Virology, Hamburg, Germany. To prevent contamination of guinea pigs from experimentators, this room was only entered with special double filter FFP3 masks. Guinea pigs were anesthetized intramuscularly (i.m.) in the hind leg with ketamine (30 mg/kg) and xylazine (2 mg/kg). When reflexes were absent under narcosis, guinea pigs were inoculated intranasally (i.n.) with a 300 µl inoculum containing 5*10^5^ p.f.u. of virus diluted in 1x PBS. Sentinel animals were mock inoculated with 1x PBS only. To test contact transmission, one infected guinea pig was placed in the same cage as a sentinel according to previously described protocols [15]. Nasal washes were performed with 1x PBS with 0.2% BSA and 1% penicillin/streptomycin (Sigma-Aldrich) on days 1, 3, 6 and 9 post infection (p.i.) to assess the potential for virus transmission.

### Immunohistochemistry

To investigate histopathological changes in the respiratory tract of mice and guinea pigs upon H7N7 influenza A virus infection, organs were processes at days 3 and 6 p.i. as described before [16]. Briefly, fixed tissues (in 4% Paraformaldehyde; Omnilab) were thin-sectioned, deparaffinised and exposed to rabbit anti-H7N1 serum (1:2000). As a secondary antibody, a biotin-conjugated anti-rabbit antibody (1:200; Jackson ImmunoResearch) was used and thin-sections were counterstained with hematoxylin.

### Ethics statement

All animal experiments were performed at the Heinrich Pette Institute, Leibniz Institute for Experimental Virology in Hamburg, Germany and were approved by the responsible German authorities (Behörde für Gesundheit und Verbraucherschutz, Fachbereich Veterinärwesen, Hamburg) and conducted according to the FELASA guidelines of animal welfare.

### Statistical analysis

Statistical significance was determined with GraphPad Prism 5 v.5.03 (Graphpad Software, Inc.) using the Student’s *t*-test. Statistical significance was defined as *P *< 0.05 (* *P *< 0.05, ** *P *< 0.01, *** *P *< 0.001).

### Sequence surveys

All available H7 protein sequences from ﬂuDB [17] and GISAID (https://www.gisaid.org/) (an acknowledgment spreadsheet of all laboratories contributing to GISAID is available upon request) was downloaded on 5 April 2019 and an alignment obtained by using the MAFFT algorithm [18] implemented in the Geneious package [19]. All duplicated, truncated, or gapped sequences were eliminated to subsequent analysis. In particular, we excluded in the alignment all those entries with identical virus strain names and identical sequences. Sequence positions include the signal peptide region.

## Results

### H7 HA mammal-adapted mutations increase virulence in mice

In order to assess viral markers that may cause increased mammalian virulence and potential mammal-to-mammal transmission, we compared an H7N7 HPAIV (SC35) [10] to its ferret-adapted variant (SC35F, H7N7) [11]. The SC35 virus is highly pathogenic in chicken but low pathogenic in mice and ferrets [10,11]. In contrast, the SC35F virus obtained by serial lung-to-lung passages in ferrets, maintained its pathogenicity in chickens but became additionally lethal in ferrets [11].

**Figure 1.**
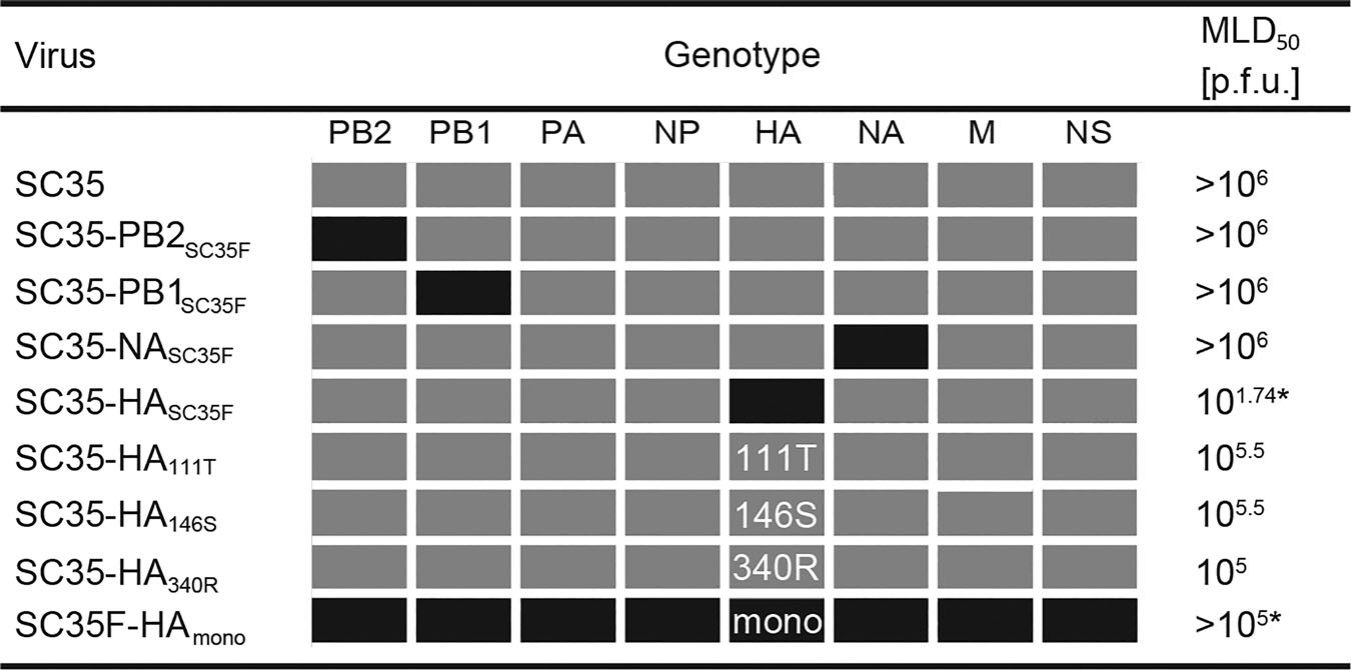
**Virulence of SC35 and SC35F recombinant viruses in mice.** BALB/c mice were inoculated intranasally with 10^1^–10^6^ p.f.u. of SC35 and SC35F recombinant viruses, respectively. Control mice were mock infected with PBS (*n *= 5). Boxes refer to the origin of the viral gene segments of SC35 (grey) or SC35F (black). The viral segments PA, NP, M and NS are identical in SC35 and SC35F. For single point mutations in HA in the SC35 genetic background, SC35F specific amino acids are indicated as 111T, 146S and 340R. Mono indicates the presence of a monobasic cleavage site in contrast to the multi-basic cleavage site of SC35 and SC35F. *indicates that in these settings the highest virus infection dose used was 10^5^ p.f.u. representing the highest virus stock dose yielded.

In this study, we sequenced the entire SC35F genome and identified six amino acid exchanges compared to the parental SC35 virus (Table 1). These mutations were localized in the genes encoding for the viral polymerase complex (PB1 L13P, PB2 H357N) as well as the viral surface proteins (HA I111T, A146S, G340R, and NA A328S). To analyze the impact of the individual gene segments as well as mutations on pathogenicity, we generated single-gene reassortant viruses (SGRs) as well as single-point mutant viruses (SPMs) containing SC35F-specific genes or single-point mutations, respectively, in the SC35 genetic backbone. First, we assessed the virulence of these recombinant viruses in mice. The mouse-lethal-dose-50 (MLD_50_) was not affected upon infection with SGRs containing the viral polymerase subunits PB2 or PB1 (SC35-PB2_SC35F_, SC35-PB1_SC35F_) or the NA surface gene (SC35-NA_SC35F_) compared to the parental SC35 (Figure 1). However, virulence was increased >3-times compared to SC35 upon infection with SPMs containing SC35F-specific single mutations in HA (SC35-HA_111T_, SC35-HA_146S_, SC35-HA_340R_). It is important to note that the HA G340R mutation present in the SC35-HA_340R_ virus confers a tetra-basic cleavage site in the hemagglutinin of SC35 that possesses a tri-basic cleavage site [10]. However, combination of the two single SC35F-specific HA amino acids 111T and 146S with the tetra-basic HA cleavage site in the recombinant SC35-HA_SC35F_ virus resulted in a dramatic increase in virulence in mice by up to ∼18.000-times compared to parental SC35 infection. In line, replacing the tetra-basic cleavage site by a monobasic cleavage site (SC35F-HA_mono_) resulted in reduced virulence in mice. These data show that a tetra-basic cleavage site combined with the two H7 mutations I111T and A146S are the major determinants of H7 AIV pathogenicity in mice.

**Table 1.** Amino acid differences between SC35 and SC35F.

	Genotype					
	PB2	PB1	HA			NA
aa position	357	13	111	146	340	328
SC35	H	L	I	A	G	A
SC35F	N	*P*	T	S	R	S

aa: amino acid.

### H7 HA mammal-adapted mutations increase pulmonary replication efficiency and pathogenicity in mice

**Figure 2.**
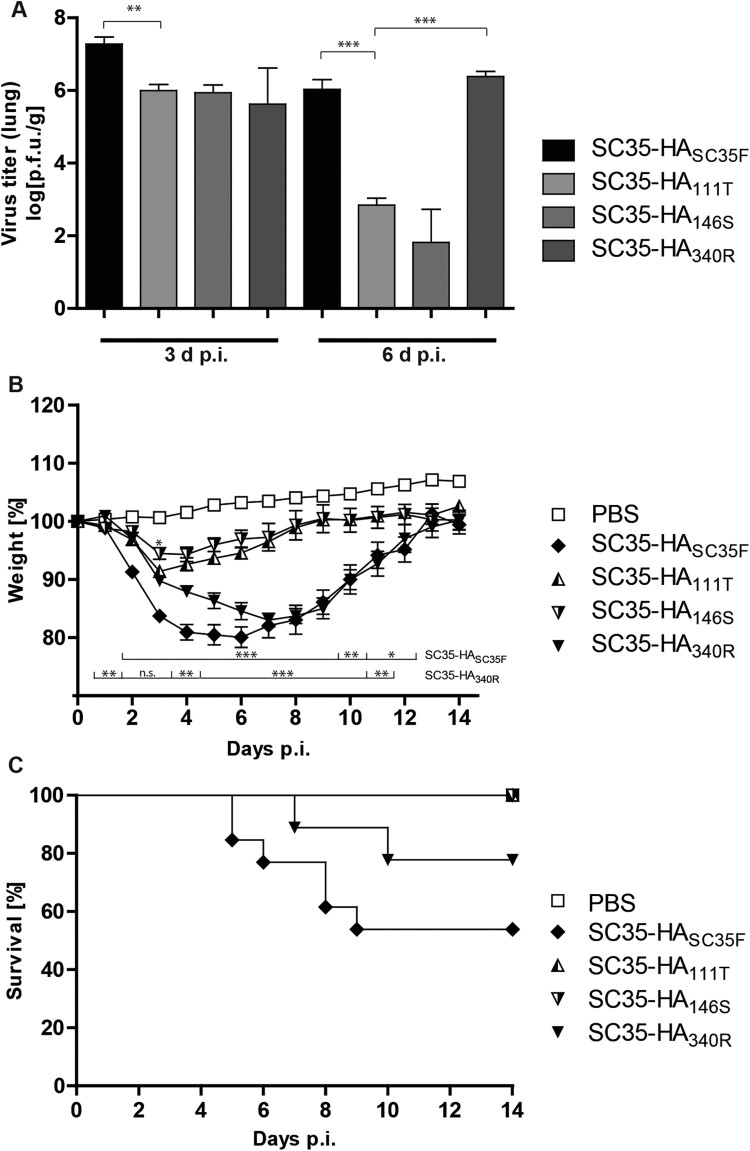
**Lung titers and morbidity in H7 SGR and SPM virus infected mice.** BALB/c mice were inoculated intranasally with 10^5^ p.f.u. SC35-HA_SC35F_ (filled diamond), SC35-HA_111T_ (triangle, half filled), SC35-HA_146S_ (upside down triangle, half filled) and SC35-HA_340R_ (filled upside down triangle). (A) From each group (*n *= 3) mice were sacrificed on 3 and 6 d p.i., lungs were harvested and viral titers of the lung homogenates were determined via plaque assay on MDCK cells. SC35-HA_SC35F_ (black), SC35-HA_111T_ (bright grey), SC35-HA_146S_ (grey) and SC35-HA_340R_ (dark grey). (B) Weight loss of PBS mock infected mice (*n = 19*), of SC35 (*n = 5*), SC35-HA_SC35F_ (*n *= 14), SC35-HA_I111T_ (*n *= 11), SC35-HA_A146S_ (*n *= 11) and SC35-HA_G340R_ (*n *= 15) infected BALB/c mice was recorded for 14 days and plotted as percentages of the original weight from day 0. (C) Survival of the in (A) shown BALB/c mice was recorded for 14 days and depicted in a survival graph.

To unravel the impact of single SC35F-specific HA mutations on replication efficiency in the murine lung, we infected mice with the SPMs SC35-HA_111T_, SC35-HA_146S_ and SC35-HA_340R_, respectively, and compared to the SGR SC35-HA_SC35F_ infection. Virus load in the lung was highest upon infection with SC35-HA_SC35F_ virus on days 3 and 6 p.i. compared to the SPMs SC35-HA_111T_ and SC35-HA_146S_ (Figure 2(A)). Virus titers upon infection with SC35-HA_340R_ were comparable to SC35-HA_SC35F_ on day 6 p.i. unlike other SPMs. Elevated virus titers in the lung generally correlated with increased weight loss and reduced survival in mice (Figure 2(B and C)). Highest weight loss was detected in SC35-HA_340R_ and SC35-HA_SC35F_ infections in contrast to SC35-HA_111T_ and SC35-HA_146S_ infections. However, lethality was highest among SC35-HA_SC35F_ infected mice. Taken together, a tetra-basic HA cleavage site and the H7 mutations I111T and A146S constitute the major determinants of H7 AIV pulmonary virus replication efficiency and pathogenicity in mice.

### H7 HA mammal-adapted mutations contribute to pneumonia in mice

**Figure 3.**
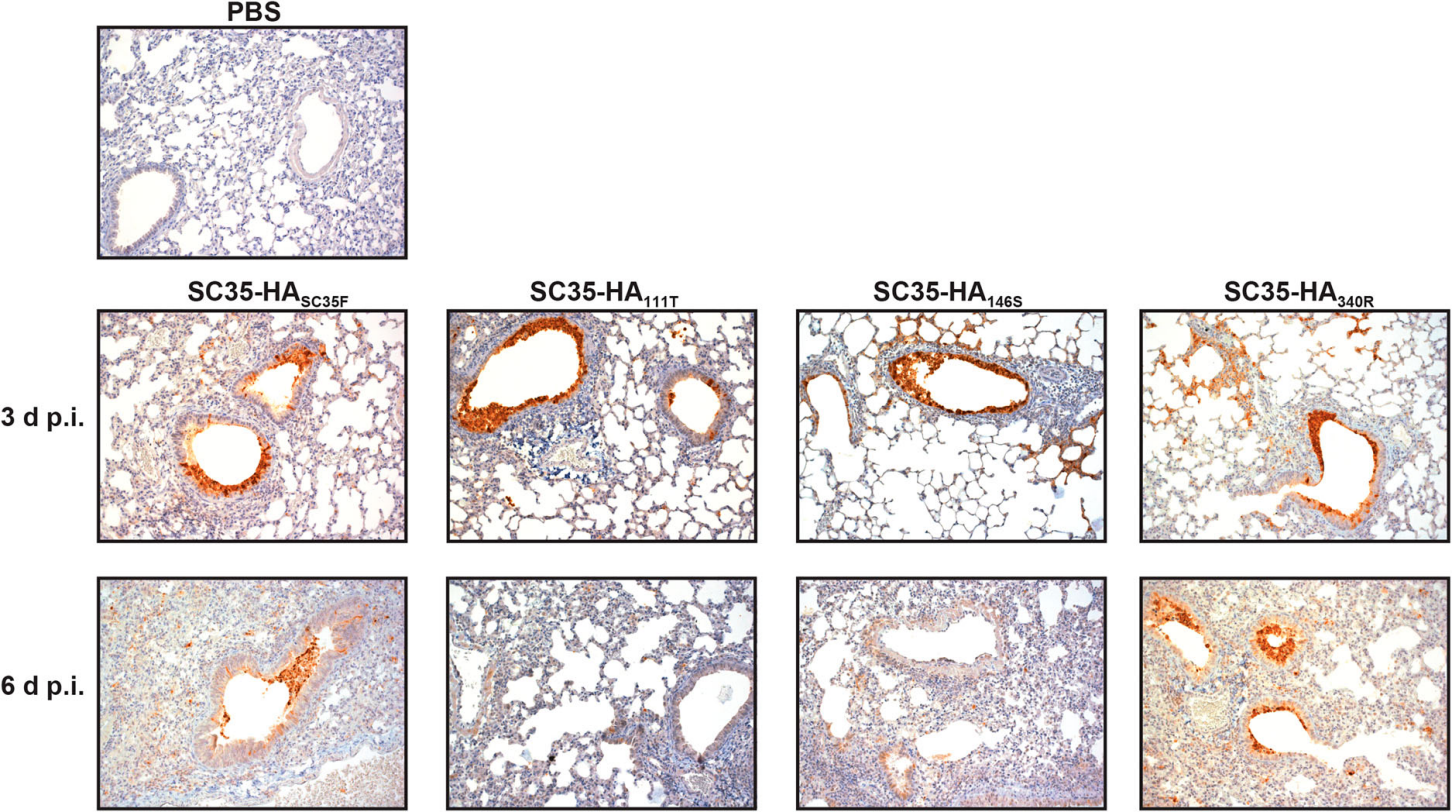
**Lung tropism of H7 SGR and SPM virus infected mice.** BALB/c mice were inoculated intranasally with 10^5^ p.f.u. of SC35-HA_SC35F_, SC35-HA_111T_, SC35-HA_146S_ and SC35-HA_340R_, respectively. On day 3 and 6 p.i. mice were sacrificed and lungs were harvested for histopathological investigation. IHC stainings were performed using polyclonal serum against H7 influenza virus and counterstaining performed with hematoxylin.

We further assessed the impact of mammalian pathogenicity relevant mutations in H7 on lung pathology in mice. As expected, SC35-HA_SC35F_ infection resulted in severe pneumonia with destructed and highly infiltrated alveoli with mononuclear cells. High signals of virus antigen positive cells were detected in the bronchiolar epithelium but also in the alveolar space (Figure 3). Infection with the respective SPM viruses SC35-HA_111T_, SC35-HA_146S_, and SC35-HA_340R_ showed similar high-level viral antigen positive bronchial epithelia as well as virus-positive cells within alveoli. However, alveolar infiltration and destruction was most severe upon infection with SC35-HA_SC35F_. These data show that a multibasic cleavage site in H7 hemagglutinin combined with the HA mutations HA I111T and A146S mediate severe pneumonia in mice.

### H7 HA mammal-adapted mutations contribute to inflammatory cytokine and chemokine responses in the murine lung

**Figure 4.**
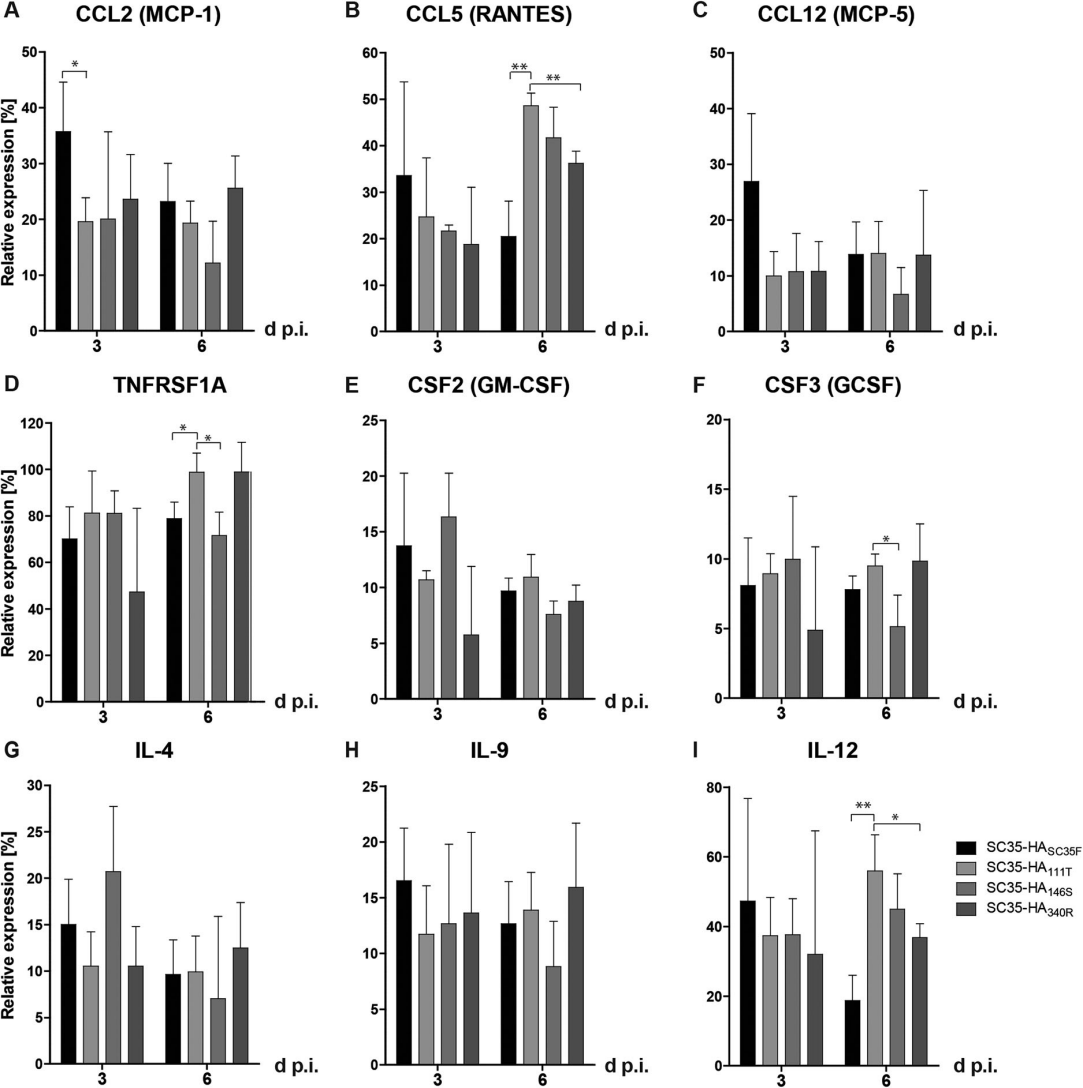
**Cytokine response in the lungs of H7 SGR and SPM virus infected mice.** Cytokine levels were detected in lung homogenates upon infection with SC35-HA_SC35F_ (black), SC35-HA_111T_ (bright grey), SC35-HA_146S_ (grey), SC35-HA_340R_ (dark grey) on days 3 and 6 p.i.. (A) Expression levels of the CC-chemokine ligand 2 (CCL2, MCP-1), (B) CCL5 (RANTES), (C) CCL12 (MCP-5), (D) TNF receptor superfamily member 1A (TNFRSF1A), (E) colony-stimulating factor CSF2 (GM-CSF), (F) CSF3 (GCSF), (G) IL-4, (H) IL-9 and (I) IL-12 were measured in lung homogenates of infected BALB/c mice (*n *= 3). The relative expression levels were calculated in relation to the positive control set to 100%.

Next, we assessed the impact of SC35F-specific HA mutations (Table 1) on inflammatory cytokine responses in the lung. Herein, we analyzed expression levels of key cytokines and chemokines upon respiratory viral infection. In general, SC35-HA_SC35F_ infection displayed the overall highest cytokine/chemokine levels assessed compared to the SPMs SC35-HA_111T_, SC35-HA_146S,_ or SC35-HA_340R_ at 3 days p.i. (Figure 4). At day 6 p.i., TNFRSF1A, CCL5 and IL-12 levels were highest in SPM SC35-HA_111T_, SC35-HA_146S_ and SC35-HA_340R_ infections compared to SC35-HA_SC35F_. These data suggest that all three mutations within the viral H7 HA (I111T, A146S, G340R) of the ferret-adapted H7 HPAIV contribute to high-level inflammatory cytokine response in the murine lung.

### Mammal-adapted H7 HA and PB1 mutations mediate contact transmission in guinea pigs

**Figure 5.**
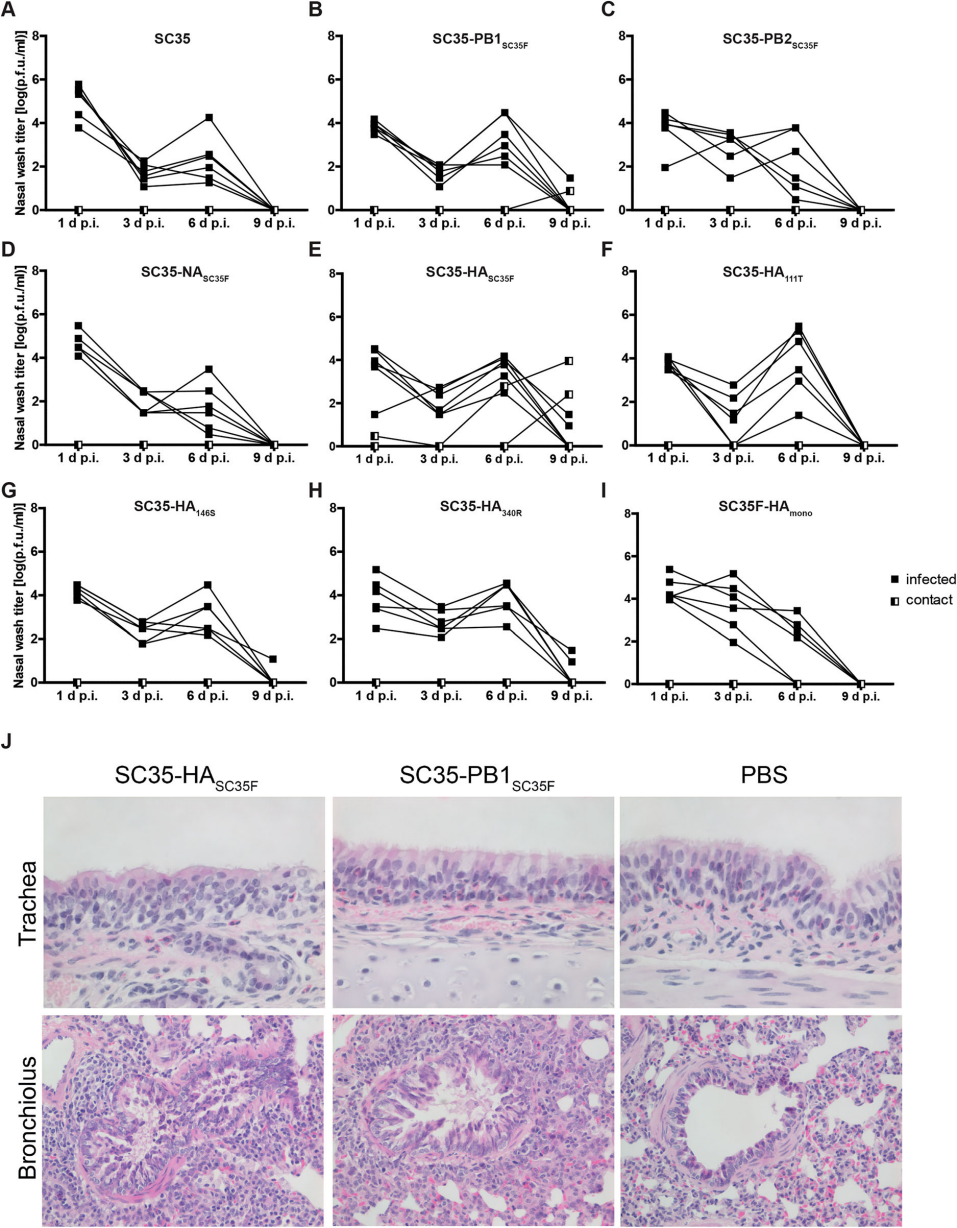
**Contact transmission of SC35 and SC35F recombinant viruses in guinea pigs.** Donor Hartley guinea pigs (filled squares; *n *= 6) were experimentally inoculated intranasally with 5*10^5^ p.f.u. of (A) SC35, (B) SC35-PB1_SC35F_, (C) SC35-PB2_SC35F_, (D) SC35-NA_SC35F_, (E) SC35-HA_SC35F_, (F) SC35-HA_111T_, (G) SC35-HA_146S_, (H) SC35-HA_340R_ and (I) SC35F-HA_mono_ recombinant viruses. Sentinel guinea pigs were introduced into the same cage, allowing direct contact between the animals (half-filled squares; *n *= 2 (A, C, D, F, G, H, I); *n *= 6 (B and E were repeated independently and results could be confirmed)). To obtain viral titers in the upper respiratory tract, nasal washes of sentinel and donor animals were determined on days 1, 3, 6 and 9 p.i. by plaque assay on MDCK cells. (J) Hematoxylin staining of the trachea and bronchioles of SC35-HA_SC35F_ and SC35-PB1_SC35F_ infected guinea pigs.

We then assessed the impact of mammalian-adapted mutations in SC35F on viral contact transmission in the guinea pig model. All infected donor guinea pigs, irrespective of the recombinant virus used, presented virus titres in nasal washes on days 1, 3 and 6 p.i. (Figure 5). Virus replication could be detected in all animals infected with SC35 or respective SGR and SPM recombinant viruses (Figure 5(A-I)). However, prolonged virus replication with detectable virus titers even as late as day 9 p.i. was observed only in animals infected with SC35-PB1_SC35F_ (1/5 donors), SC35-HA_SC35F_ (2/5 donors), SC35-HA_146S_ (1/4 donors) and SC35-HA_340R_ (2/6 donors) recombinant viruses (Figure 5(B, E, G and H)). Sentinel animals placed in the same cage of the infected donors remained mainly virus-negative except upon SC35-PB1_SC35F_ (1/6 sentinels) and SC35-HA_SC35F_ (2/6 sentinels) infections (Figure 5(B and E)). The positive contact transmission occurrence in sentinels upon exposure to SC35-PB1_SC35F_ and SC35-HA_SC35F_ infected donors was repeated and verified independently resulting in the same transmission efficiencies indicated above (data not shown). Interestingly, the tetra-basic HA cleavage site alone in SC35-HA_340R_ was not sufficient to mediate contact transmission (Figure 5(H)). However, infection with SC35F-HA_mono_ did also not result in virus replication positive sentinels (Figure 5(I)). This suggests that the combination of all three HA adaptive sites is required for contact transmission between guinea pigs. In line with increased replication kinetics, lungs of guinea pigs infected with SC35-HA_SC35F_ and SC35-PB1_SC35F_ showed more infiltrated mononuclear cells compared to PBS treated animals (Figure 5(K)). These findings suggest that increased contact transmission from mammal-to-mammal requires mutations in PB1 L13P and in HA (I111T, A146S and G340R), albeit we cannot entirely exclude contact transmission in the other virus mutants due to low numbers of sentinel animals.

### Mammal-adapted mutations in PB1 and H7 HA mediate increased viral polymerase activity and pH tolerance

**Figure 6.**
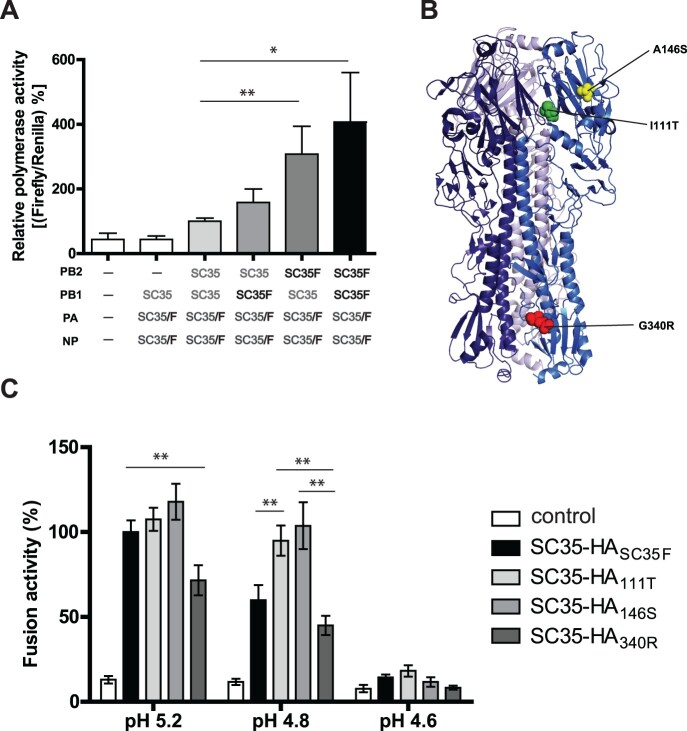
**HA fusion activity of H7 SGR and SPM viruses.** (A) Viral polymerase activity was measured of SC35F-specific mutations in the SC35 background. SC35 polymerase activity was set to 100%. As a background control, vRNPs were transfected omitting the PB2 subunit. (-) indicates that empty vectors were transfected as a negative control. The results shown are derived from five independent experiments with SEM. The significance was calculated using the Student's *t*-test comparing to SC35 RNP activity (*p < 0.1, *** *p *< 0.001). (B) Model of H7 crystal structure showing the H7 adaptive mutations (I111T in green, A146S in yellow and G340R in red; PyMol Molecular Graphics System – Protein Data Base ID: 4DJ6). (C) Cell-to-cell fusion assay. 293T cells were co-transfected with SC35-HA_SC35F_, SC35-HA_111T_, SC35-HA_146S_ and SC35-HA_340R_ expression plasmids and pGAL4-VP16 plasmid, respectively and co-cultured with 293T cells transfected with pGal5-luc plasmid. Eight hours later, co-cultured cells were treated with cell culture medium of different pH values (pH 5.2 – pH 4.6). Here, results of at least three independent experiments in triplicates are shown with SEM.

In order to shed light on the molecular basis involved in PB1 L13P and H7 HA I111R, A146S and G340R-mediated increase in contact transmission of AIV from guinea pig-to-guinea pig, we further investigated potential molecular functions. First, we measured viral polymerase activity in human cells as a known contributor to replicative fitness, pathogenicity and contact transmission in mammals [10,15,20,21]. The SC35F-specific PB1 L13P mutation caused an increase in viral polymerase activity in human cells compared to the SC35 polymerase complex (Figure 6(A)). Viral polymerase activity was also increased upon introduction of the SC35F-specific mutation in PB2 H357N. However, highest polymerase activity was measured when combining the two SC35F-specific mutations in PB1 and PB2. Thus, the SC35F polymerase activity is higher than the SC35 polymerase activity by approximately 4-times. We then analyzed whether the adaptive mutation in NA A328S might affect viral neuraminidase activity. No differences were observed between SC35-NA and SC35F-NA to sediment from hemagglutinated erythrocytes or to cleave fluorescing substrates (Supplementary Figure). Next, we assessed whether H7 mammalian-adaptive mutations in H7 HA may alter receptor binding specificity from avian-like α2,3-SA to human-like α2,6-SA receptor binding as a crucial determinant of pandemic spread [22]. However, all HA recombinant SGR (SC35-HA_SC35F_) and SPM (SC35-HA_111T_, SC35-HA_146S_, SC35-HA_340R_) viruses presented a strong binding preference for avian-like α2,3-SA receptors similar to the parental SC35 AIV (Table 2). As expected, control viruses displayed either avian-like α2,3-SA (H5N1) or human-like α2,6-SA (H3N2) receptor binding properties. Thus, mammalian-adapted mutations in H7 do not alter receptor specificity. Next, we assessed whether adaptive H7 mutations localized on the protein surface (Figure 6(B)) might affect viral membrane fusion in infected human cells as a crucial determinant of mammalian interspecies transmission [20,21]. In a cell–cell fusion assay, we detected that the adaptive mutations in HA I111T and A146S increase pH tolerance at pH5.2 similar to cells transfected with the SC35-HA_SC35F_ plasmid containing all three adaptive HA mutations (Figure 6(C)). However, at pH5.2, cells transfected with the SC35-HA_340R_ plasmid containing the multibasic cleavage site were not able to increase cell fusion compared to the other adaptive HA sites. At pH 4.8, cell fusion activity was even highest in cells transfected with plasmids containing the HA I111T and A146S mutations compared to SC35-HA_340R_ or even SC35-HA_SC35F_. At pH 4.6 only residual fusion activity was detected with no significant differences among the HA mutants. Thus, HA mutations I111T and A146S confer fusion activity at lower pH, which is a hallmark of viral transmissibility [20,21,23,24], compared to the HA multibasic cleavage site mutant HA 340R. Finally, increased viral polymerase activity and HA stability correlate with increased H7 HPAIV contact transmission.

**Table 2.** Sialic acid binding properties of H7 recombinant viruses.

Virus	Untreated TRBCs	VCNA treated TRBCs	α-2,3 SA TRBCs	α-2,6 SA TRBCs
SC35	64	0	64	0
SC35-HA_SC35F_	64	0	64	0
SC35-HA_111T_	64	0	64	0
SC35-HA_146S_	64	0	64	0
SC35-HA_340R_	64	0	64	0
H3N2 control	64	0	0	64
H5N1 control	64	0	128	0

Results are shown as hemagglutination units. Data are representative of 3–6 independent assays. VCNA treated TRBCs: TRBCs were treated with neuraminidase of *Vibrio cholerae* (VCNA) and the elimination of the sialic acids (SA) was assessed as a control. α2,3/α2,6 SA TRBCs: after VCNA treatment, the TRBCs were resialylated using either α2,6-sialyltransferase from *Photobacterium damsela* or α2,3-Sialyltransferase from *Pasteurella multocida*. As additional controls, we used viruses with known sialic acid binding preferences. As a control for α2,6-linked SA binding, A/Netherlands/213/03 (H3N2) was used and as a control for α2,3-linked SA binding an H5N1 SGR was used containing the HA segment of A/Vietnam/11/94 (H5N1) with a monobasic cleavage site in the A/PR/8/34 (H1N1) virus backbone as described before [16].

### Prevalence of mammal-adapted H7 HA mutations in circulating H7 strains

To survey the prevalence of the H7 HA mutations I111T and A146S in circulating IAV strains, we downloaded all available H7 HA peptide sequences from Genbank [17] and GISAID (https://www.gisaid.org/) and performed alignments. Among 4602 total HA sequences analyzed, 3152 entries carried at the amino acid position 111 V, but 12 HA sequences harbored 111I, and 1312 HA sequences possessed 111T (Table 3). Whereas an I residue was found in some mammalian strains (feline and SC35M), T was predominantly found in avian strains (Table 4). At HA position 146, 4438 entries carried A, whereas S was found in only 63 entries representing mostly avian strains (Tables 3 and 4). Among the H7N9 Asian group (1980 entries), almost all strains contained 111V (1970) and 146A (1977). Taken together, the SC35F amino acids 111T and 146S in H7 HA were found predominantly in avian strains from different hosts (Galliformes, Anseriformes, and Charadriiformes).

**Table 3.** Amino acids and frequencies at HA 111 and 146.

Position	Frequencies
111	V: 68% (3152), T: 29% (1321), A: 1% (46), S: 1% (35), D: 1% (29), I: 0% (12), M: 0% (6), –: 0% (1)
146	A: 96% (4438), T: 2% (96), S: 1% (63), –: 0% (3), D: 0% (2)

Total: 4602 HA sequences

**Table 4.** Amino acid frequencies at positions HA 111 and 146 in different host species.

111V			111I			111T		
Host	Found	Fraction [%]	Host	Found	Fraction [%]	Host	Found	Fraction [%]
Human	1318	41.8	Human	1	8.3	Human	4	0.3
Mammal	1	0.0	Mammal	5	41.7	Mammal	4	0.3
av, GF	935	29.7	av, GF	1	8.3	av, GF	321	24.3
av, af	528	16.8	av, af	5	41.7	av, af	664	50.2
av, unknown	10	0.3				av, unknown	72	5.5
av	67	2.1				av	8	0.6
av, cf	3	0.1				av, cf	139	10.5
unknown	289	9.2				unknown	109	8.3
Total:	3151	100.0	Total:	12	100.0	Total:	1312	100.0
**146A**			**146S**					
**Host**	**Found**	**Fraction [%]**	**Host**	**Found**	**Fraction [%]**			
Human	1324	29.8	Human	1	1.6			
Mammal	37	0.8						
av, GF	1173	26.4	av, GF	49	77.7			
av, af	1211	27.3	av, af	11	17.5			
av, unknown	84	1.9						
av	69	1.6	av	1	1.6			
av, cf	142	3.2						
unknown	398	9.0	unknown	1	1.6			
Total:	4438	100.0	Total:	63	100.0			

av – avian, GF – Galliformes, af – Anseriformes, cf – Charadriiformes

## Discussion

In this study, we took advantage of a ferret adaptation model to study molecular markers of H7 HPAIV mammalian adaptation and transmission. The ferret-adapted H7N7 HPAIV SC35F was obtained in 1995 from ferrets by serial lung-to-lung passages of the H7N7 SC35 HPAIV that is highly pathogenic in chicken but low pathogenic in mice [11].

Here, we identified two mutations in the H7 hemagglutinin (I111T and A146S) combined with a tetrabasic cleavage site (G340R) to increase H7N7 virulence in mice. However, contact transmission from guinea pig-to-guinea pig, assessed by putting the sentinel animal into the same infected donor cage, required an additional mutation in PB1 L13P combined with the adaptive H7 mutations. None of the single-gene reassortants in the SC35 genetic backbone containing a single gene segment of SC35F was able to show airborne transmission if sentinel guinea pigs were placed into adjacent cages of infected donor animals (data not shown). However, due to the classification of the SC35F strain as a BSL-4 agent, we were not able to test the entire SC35F strain. Thus, it remains open whether SC35F would be able to mediate airborne transmission.

Another limitation of the study is that SC35F as a ferret-adapted variant was not tested in ferrets, which are usually more sensitive to airborne transmission than guinea pigs [15,16]. Despite these limitations, it is interesting to compare the results obtained from different mammalian adaptation models that allow accelerated virus evolution and thereby rapid identification of pandemic risk factors.

We have previously shown that adaptation of SC35 to mice revealing SC35M [11], resulted mainly in mutations in the viral polymerase complex, such as PB2 D701N, PB1 L13P and NP N319K that increased viral polymerase activity in human cells and virulence in mice [10]. In parallel, adaptation of SC35 to ferrets as another mammalian animal model also led to mutations in viral polymerase subunits (PB1 L13P and PB2 H357N) that increased viral polymerase activity in human cells [10,25]. Additionally, ferret adaptation revealed mutations in HA (I111T, A146S and G340R) that increase virulence in mice and contact transmission in guinea pigs. This finding corresponds to studies on H5N1 HPAIV that were adapted to ferrets [20,21]. The virulence-increasing HA mutations I111T and A146S were found predominantly in strains isolated from different avian host groups whereas the PB1 exchange L13P is prevalent in nearly 100% of PB1 sequences analyzed irrespective the virus host (results not shown). This observation corresponds to the finding that a 1918-like virulent virus could be reconstituted from gene segments distributed among different unrelated avian strains [26]. Therefore, prevalence of mutations, such as the identified H7 exchanges plus emerging combinations thereof should be subjected to continuous surveillance.

Adaptation of an H5N1 HPAIV to ferrets resulted in airborne transmission from ferret-to-ferret and was mediated by several mutations, such as mutations in the polymerase subunits PB2 (E627K), a known marker of elevated viral polymerase activity in human cells, as well as in PB1 (H99Y, I368V), NP (R99K, S345N) combined with mutations in the H5 hemagglutinin (H103Y, T156A, Q222L, G224S) [20]. The H5 adaptive mutations Q222L and G224S were associated with enhanced virus binding to human-like α2,6-linked sialic acid containing receptors [20] that are expressed in the upper respiratory tract in humans [27,28]. Infection of the upper respiratory tract is believed to be essential for pandemic influenza viruses in order to spread efficiently from human-to-human [27,28].

Thus, adaptation to mice and ferrets reveal mutations in the viral polymerase complex that increase replicative fitness in human cells albeit the identified mutations in the polymerase complex may vary. This is in line with the idea of convergent virus evolution [29], proposing that similar functions obtained in an adaptive environment might be acquired by different mutations [9,30–32]. Thus, the adaptive feature, in this case increased viral polymerase activity, seems to be key in animal models, such as mice, guinea pigs and ferrets in mediating increased pathogenicity.

However, features of increased transmission that require mutations in HA seem not to be acquired in mice but require animal models that allow animal-to-animal transmission, such as guinea pigs or ferrets. The underlying reason could be the different receptor expression profile in the ferret respiratory tract resembling more the human airway expressing α2,6-linked sialic acid containing receptors predominantly [33] in contrast to the murine respiratory tract carrying avian-like α2,3-linked sialic acid containing receptors solely [34]. The polymerase activity increasing mutations in this study were shown before to increase viral polymerase activity in human cells (PB1 L13P and PB2 H357N) and virulence in mice [10,35]. The PB2 357 position was shown to play a key role in Cap-binding [25], which might explain how the PB2 H357N mutations could increase the polymerase activity. The herein identified two new H7 mutations (I111T and A146S) that contribute to increased H7N7 pathogenicity in mice and contact transmission in guinea pigs do not alter receptor specificity. All SC35F HA mutants preferentially bound to avian-like α2,3-linked sialic acid containing receptors. However, both H7 adaptive mutations HA mutations I111T and A146S conferred fusion activity at lower pH, which is a hallmark of viral transmissibility [20,21,23,24].

Thus, our study highlights that mutations in H7 hemagglutinin that shift the HA stability to lower pH play a key role in H7 HPAIV pathogenicity and contact transmission. They should therefore be included in the pandemic risk assessment of emerging H7 AIV.

## Supplementary Material

Supplemental Material
