# Supplementary figures and images for: Mutations in the H7 HA and PB1 genes of avian influenza a viruses increase viral pathogenicity and contact transmission in guinea pigs
Source: Emerg Microbes Infect. 2019 Sep 10;8(1):1324–36. doi: 10.1080/22221751.2019.1663131 (PMC6746284; doi:10.1080/22221751.2019.1663131)

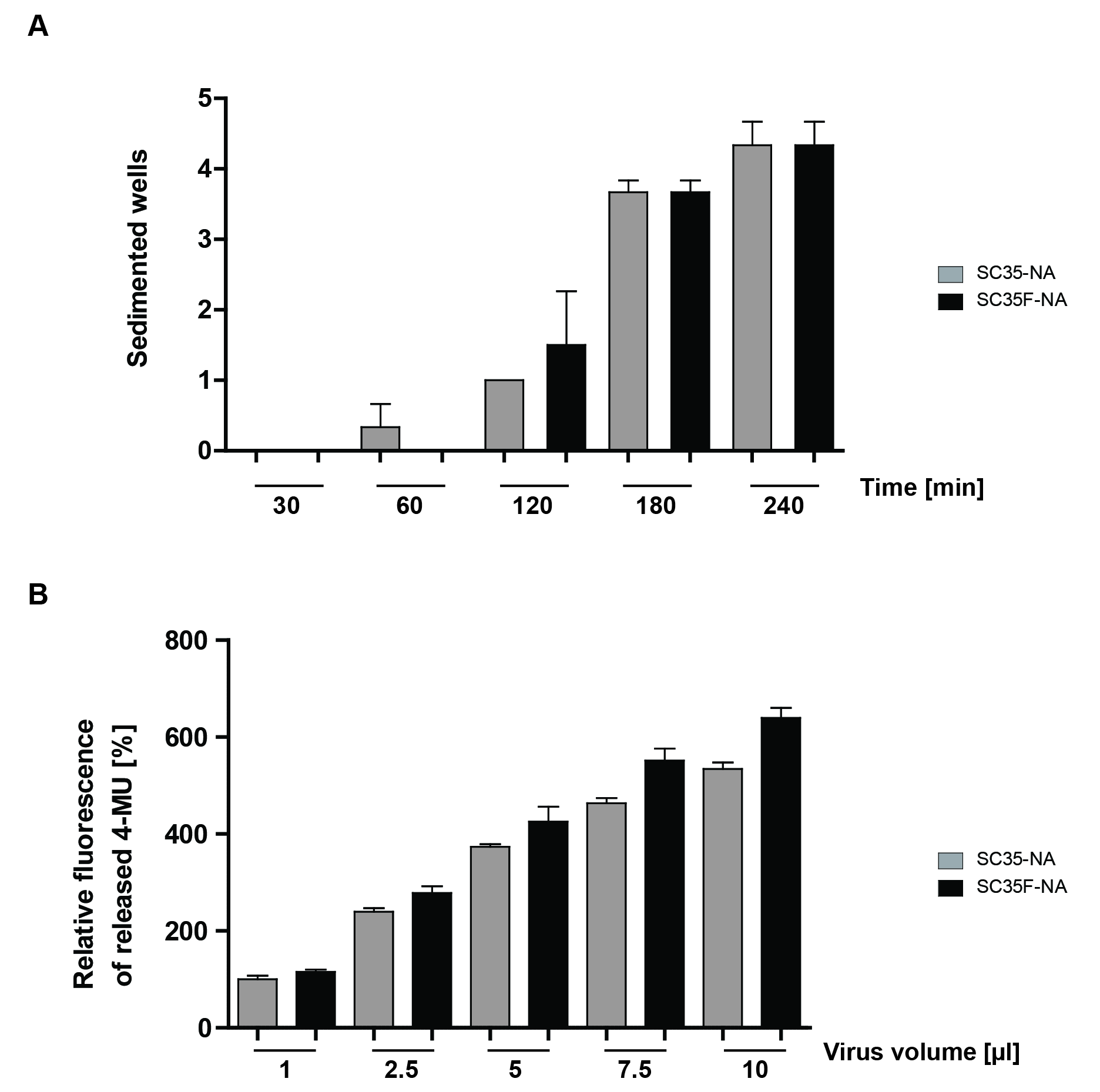

Supplement: Supplemental Material [file TEMI_A_1663131_SM0341.tif]
